# The effectiveness, reproducibility, and durability of tailored mobile coaching on diabetes management in policyholders: A randomized, controlled, open-label study

**DOI:** 10.1038/s41598-018-22034-0

**Published:** 2018-02-26

**Authors:** Da Young Lee, Jeongwoon Park, Dooah Choi, Hong-Yup Ahn, Sung-Woo Park, Cheol-Young Park

**Affiliations:** 1 Division of Endocrinology and Metabolism, Department of Internal Medicine, Kangbuk Samsung Hospital, Sungkyunkwan University School of Medicine, Seoul, Republic of Korea; 2 Division of Endocrinology and Metabolism, Department of Internal Medicine, Korea University College of Medicine, Seoul, Republic of Korea; 3 Huraypositive Inc. Sinsa-dong, Gangnam-gu, Seoul Republic of Korea; 4 Department of Statistics, Dongguk University-Seoul, Seoul, Republic of Korea

## Abstract

This randomized, controlled, open-label study conducted in Kangbuk Samsung Hospital evaluated the effectiveness, reproducibility, and durability of tailored mobile coaching (TMC) on diabetes management. The participants included 148 Korean adult policyholders with type 2 diabetes divided into the Intervention-Maintenance (I-M) group (n = 74) and Control-Intervention (C-I) group (n = 74). Intervention was the addition of TMC to typical diabetes care. In the 6-month phase 1, the I-M group received TMC, and the C-I group received their usual diabetes care. During the second 6-month phase 2, the C-I group received TMC, and the I-M group received only regular information messages. After the 6-month phase 1, a significant decrease (0.6%) in HbA1c levels compared with baseline values was observed in only the I-M group (from 8.1 ± 1.4% to 7.5 ± 1.1%, *P* < 0.001 based on a paired *t*-test). A*t* the end of phase 2, HbA1c levels in the C-I group decreased by 0.6% compared with the value at 6 months (from 7.9 ± 1.5 to 7.3 ± 1.0, *P* < 0.001 based on a paired *t*-test). In the I-M group, no changes were observed. Both groups showed significant improvements in frequency of blood-glucose testing and exercise. In conclusion, addition of TMC to conventional treatment for diabetes improved glycemic control, and this effect was maintained without individualized message feedback.

## Introduction

The incidence and prevalence of type 2 diabetes are increasing rapidly worldwide, and the disease is expected to affect 439 million adults by 2030^1^. Previous large clinical trials indicated that adequate glycemic control contributed to a reduction in both microvascular and macrovascular complications as well as mortality rates due to diabetes^2,3^. Complications from diabetes result in greater expenditure and reduced productivity. Therefore, it is a socioeconomic concern^4,5^. Adequate glycemic control is important not only as an individual health problem, but also as a challenge to healthcare systems worldwide.

However, approximately 40% of subjects with diabetes in the United States do not meet the recommended target for glycemic control, low-density lipoprotein cholesterol (LDL-C) level, or blood pressure (BP)^6^. In Korea, glycated hemoglobin (HbA1c) levels for nearly half of diabetic patients were above 7.0%^7^.

Although successful diabetes care requires therapeutic lifestyle modification in addition to proper medication^8–10^, only 55% of individuals with type 2 diabetes receive diabetes education from healthcare professionals^11^, and 16% report adhering to recommended self-management activities^9^. Multifaceted professional interventions are needed to support patient efforts for behavior change including healthy lifestyle choices, disease self-management, and prevention of diabetes complications^10^.

Digital healthcare based on information and communication technology is one approach to such intervention. The widespread distribution of mobile phones and electronic communication across socioeconomic, gender, and age groups combined with the ability to process and communicate data in real time, renders these modalities ideal platforms to create simple and effective diabetes management programs^12,13^. This technological approach is a promising opportunity to overcome time and location barriers and provide real-time individualized medical treatments^14^.

Several previous attempts for diabetes management using digital healthcare resulted in improved glycemic control^13–18^. However, other studies did not ask if these results were reproducible in other population groups, persisted after the end of the intervention, or if other clinical outcomes than HbA1c level were impacted^13,17,19^.

Therefore, the present study asked if the addition of a tailored mobile coaching (TMC) system to current primary care for diabetes management resulted in better glycemic control and other diabetes-related outcomes in adult patients with type 2 diabetes compared to standard diabetes management. In addition, the reproducibility and durability of the TMC system were assessed.

## Methods

### Study participants

Korean policyholders with type 2 diabetes were recruited from Samsung Fire and Marine Insurance (Seoul, South Korea) from October 2014 to December 2015. Two-hundred subjects were eligible based on the following inclusion criteria: age ≥19 years; smartphone user; HbA1c ≥ 6.5% within the last 3 months. Among them, 52 subjects were excluded for any of the following: currently had serious concomitant disease other than diabetes; malignancy-related histories on admission, myocardial infarction, cerebral infarction or organ transplantation; pregnant or had plans for pregnancy within 6 months; plans to participate in other clinical studies or illiteracy. Finally, 148 subjects were enrolled in this study.

All participants provided written informed consent before any study procedures were started. The trial protocol was reviewed and approved by the Institutional Review Board of Kangbuk Samsung Hospital (KBS12089) and was conducted in accordance with the Helsinki Declaration of 1975. The trial was registered with clinicaltrials.gov (Clinical trial reg. no. NCT03033407), and the date of registration is January 26, 2017.

### Study design and details of the TMC system

This study was a randomized, controlled, open-label trial, conducted from October 2014 to December 2015 in Kangbuk Samsung Hospital, Seoul, Korea, and consisted of 2 phases (Supplementary Fig. S1). In phase 1, the effectiveness of the TMC system (described in the next section) was estimated; in phase 2, the reproducibility and durability of the TMC system were evaluated. Intervention was defined as the addition of the TMC system to current diabetes care. The participants maintained their previous diabetes management under the direction of their primary care physician in outside clinics throughout this study, and the researchers were not involved with patient prescriptions.

Prior to randomization, all participants underwent baseline measurements; were equally educated regarding usual dietary, nutritional, and exercise recommendations for 2 hours at an outpatient clinic in Kangbuk Samsung Hospital; and were randomly assigned into 2 groups, Intervention-Maintenance (I-M) (n = 74) and Control-Intervention (C-I) (n = 74). For allocation of the participants, a computer-generated list of random numbers produced by statistician with no clinical involvement in the trial was used. Participants were allocated in the order in which they were registered.

The sample size in each treatment group (72 for I-M group and 73 for C-I group) afforded 80% power at a significance level of α = 0.05, considering a true mean difference of changes in HbA1c of −0.50 based on prior research of similar interventions^19^ with a 15% drop-out rate.

In phase 1 of the study, participants in the I-M group received TMC, while participants in the C-I group maintained their usual diabetes care. After 6 months, phase 2 of the study was conducted and included the subjects who agreed to participate (Supplementary Fig. S1). During the second 6-month phase 2, participants in the C-I group received TMC for diabetes management. Participants in the I-M group could also access the Switch application, but received only regular messages regarding seasonal and health information without individualized message feedback by healthcare professionals.

**Figure 1:**
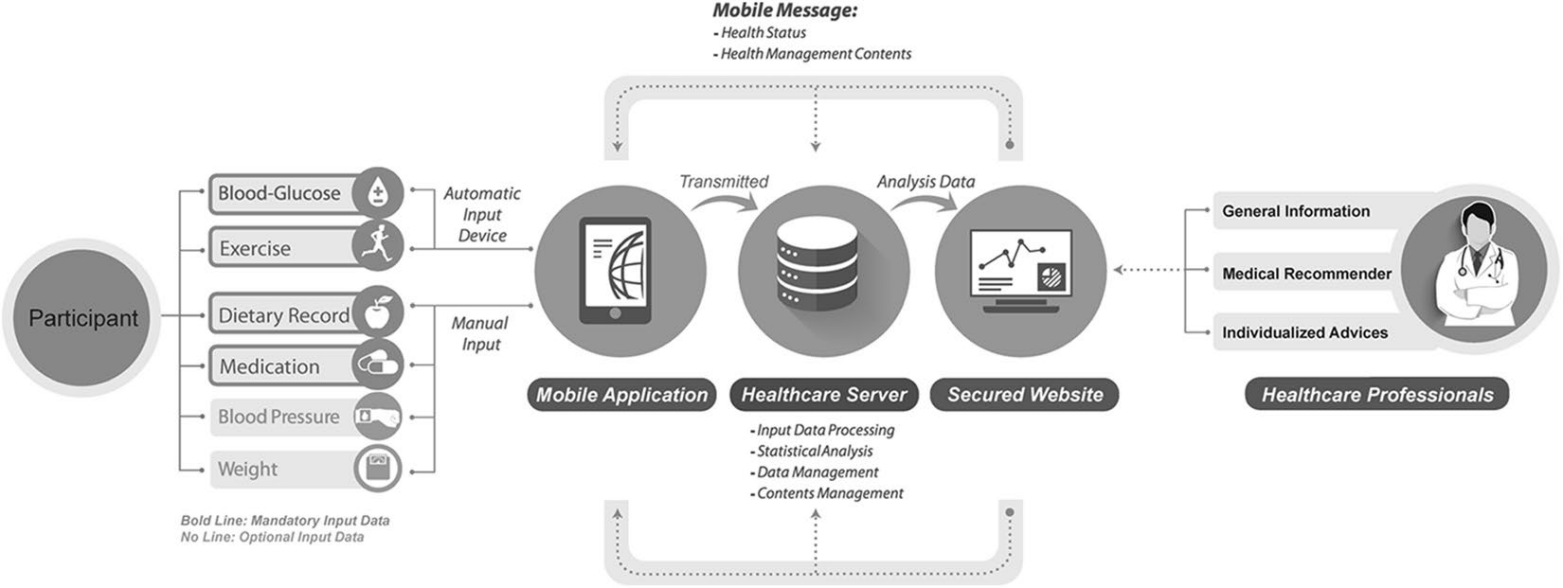
Schematic diagram of the tailored mobile coaching system.

Participants who received TMC were trained for 1 hour on how to use this service, uploaded the mobile application Switch (Huraypositive Inc, Seoul, Korea) available for both Android and iOS onto their own smartphone, and were given a Near Field Communication (NFC)-enabled glucometer (CareSens N NFC; i-SENS, Seoul, South Korea) and Bluetooth-enabled wearable fitness tracker (Fitbit flex; Fitbit Inc., San Francisco, CA, USA). As shown in Fig. 1, users could upload their lifestyle and medical information, such as self-monitoring of blood glucose (SMBG), BP, exercise, dietary record, medication record, and body weight, and set their lifestyle goals automatically presented using algorithms based on self-reported behavioral habits. At any time, users could check their data by logging into the Switch application where they could obtain information on diabetes and other metabolic diseases. The data entered in the Switch application were automatically transmitted by wireless network to the server and stored on a secure website accessible only to providers.

During the intervention period, participants received regular mobile messages and were allowed to communicate with providers *via* the Switch application. The healthcare professionals, consisting of an endocrinologist, a nurse, and a dietitian, analyzed the transmitted records and sent messages on the secured website twice a week. Message contents included an alert for the unused, weather, behavioral recommendations, education regarding diabetes, and individualized advice based on clinical practice recommendations from the American Diabetes Association and the Korean Diabetes Association^20,21^.

Communication between participants and staff was conducted mainly using application messages rather than telephone except for information regarding the study progress every 3 months and technical problems. For technical problems, participants could both send application messages and call server administrators.

### Measurements and statistical analyses

On the first visit, participants completed a self-administered questionnaire regarding demographic characteristics, social history, and other medical conditions. Smoking and drinking habits were categorized as noncurrent or current. Heights and weights were measured with subjects wearing light clothes using the same scale. The body mass index (BMI) of subjects was calculated as weight in kilograms divided by the square of height in meters. BP was measured twice using a standardized sphygmomanometer after 5 minutes of rest, and the smaller value was selected for use in the study.

Venous blood samples were collected in the morning (8–9 AM) after an overnight fast of more than 8 hours. Concentrations of HbA1c, fasting plasma glucose (FPG), total cholesterol (TC), triglycerides (TG), high-density lipoprotein cholesterol (HDL-C), and LDL-C were measured. The hexokinase method was used to test FPG concentrations. An enzymatic method was used to measure TC and TG concentrations. A homogeneous enzymatic calorimetric test was used to measure HDL-C and LDL-C levels. HbA1c was measured using the turbidimetric inhibition immunoassay. The biochemical values were measured using the Cobas Modular 6000 analyzer series (Roche Diagnostics, Basel, Switzerland). The methodology was aligned with the Diabetes Control and Complications Trial and National Glycohemoglobin Standardization Program standards^22^. The coefficients of variation for normal and abnormal values were 1.94% and 2.2% for HbA1c, 9.57% and 5.39% for cholesterol, and 7.54% and 4.14% for TG, respectively.

To assess diabetes self-management level and diabetes awareness, we used the Korean version of the Summary of Diabetes Self-Care Activities Questionnaire (SDSCA) and the Korean version of the Appraisal of Diabetes Scale (ADS). The SDSCA includes 5 subscales assessing diet (general and specific diets), exercise, blood-glucose testing, foot care, and smoking status over the past week, with higher scores indicating better self-care behaviors^23,24^. The ADS is a brief, 7-item questionnaire used to evaluate patient awareness of the psychological burden of diabetes and their understanding of diabetes management; smaller total score indicates more positive appraisal^25,26^. The Korean versions of the SDSCA and ADS have been previously validated^24,26^.

All measurements except height were repeated at 6 and 12 months. Additionally, venous sampling, BP, height, and weight were measured at 3 months. Participants were asked to visit the hospital twice, at baseline and after 12 months. Measurements at 3 and 6 months were conducted during home visits.

Data were expressed as the mean ± standard deviation (SD) or as number (proportion). We conducted a per protocol analysis. Student’s *t*-test and Chi-square test were used to examine baseline differences between the I-M and C-I groups.

The primary outcome of this study was HbA1c level. HbA1c levels in the 2 groups were compared using an independent *t*-test, and the differences before and after phases 1 and 2 within each group were analyzed using a paired *t*-test. The changes in BMI, lipid profiles, BP, and SDSCA and ADS scores, which were the secondary outcomes of this study, were analyzed using paired *t*-tests and Wilcoxon signed rank test.

Statistical analyses were performed using R version 2.14.2 (Vienna, Austria; http://www.R-project.org). A *P* value < 0.05 was considered statistically significant.

### Data availability

The data used and/or analyzed during the current study are available from Samsung Fire & Marine Insurance Company, but restrictions apply to the availability of these data, which were used under license for the current study and so are not publicly available. Data are available from the authors upon reasonable request and with permission of Samsung Fire & Marine Insurance Company.

### Ethics approval and consent to participate

All participants provided written informed consent before any study procedures were started. The trial protocol was reviewed and approved by the Institutional Review Board of Kangbuk Samsung Hospital (KBS12089) and was conducted in accordance with the Helsinki Declaration of 1975.

## Results

### Demographics and baseline characteristics of the participants

As shown in Supplementary Table S1, baseline characteristics were similar between the two groups except for current drinking habits; there were more drinkers in the C-I group.

### Phase 1 results

**Figure 2:**
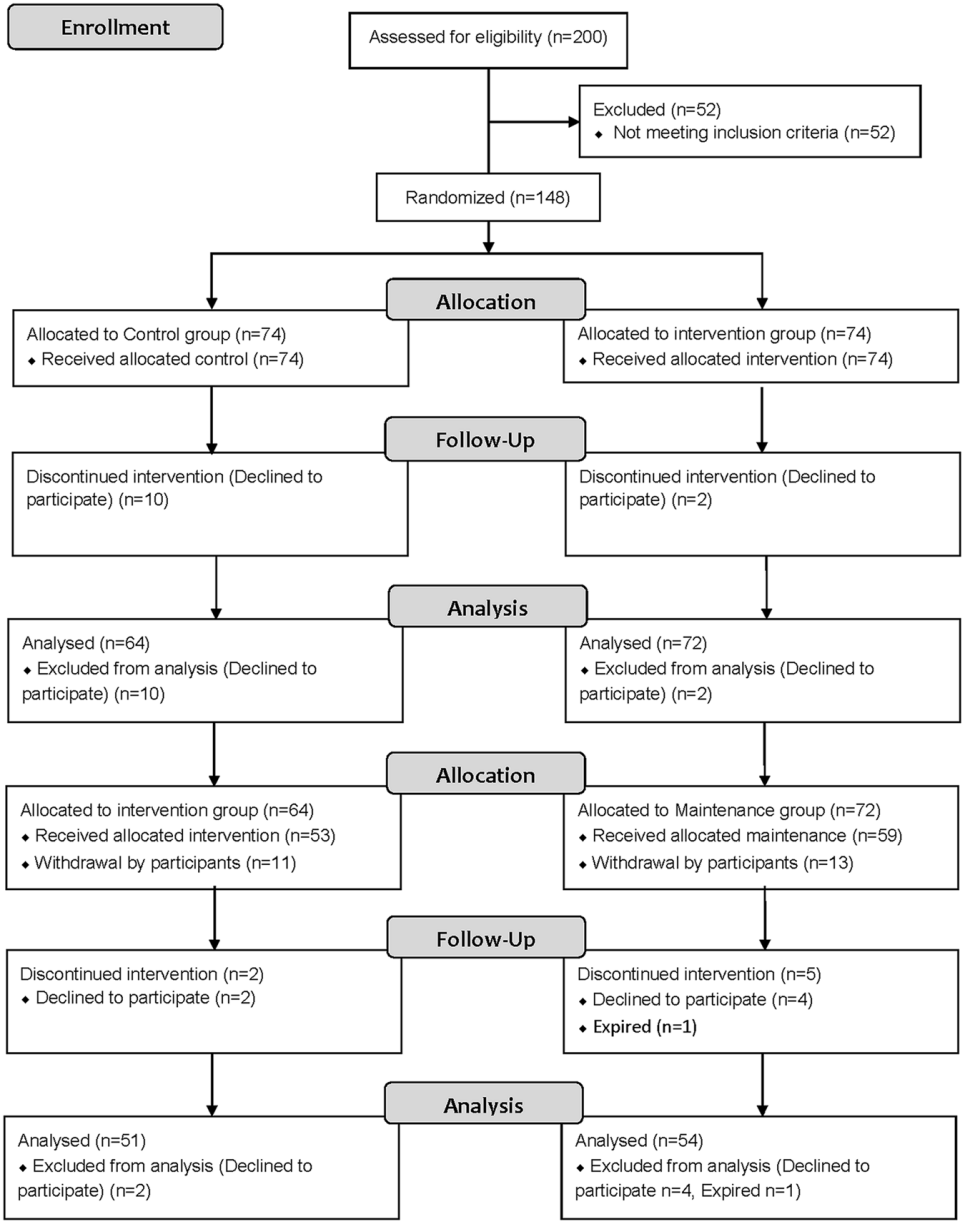
Consort flow diagram.

Among 148 participants, 136 completed phase 1 of the study (Fig. 2, the numbers of the I-M and C-I group were 72 and 64 subjects, respectively). The dropout rate was 8.1% (12 of 148 participants were lost to follow-up). During the 6-month phase 1, 72 participants of the I-M group received approximately 99.3 ± 12.5 app messages (9.5 ± 2.8 messages of tailored feedback and 89.8 ± 13.1 automated messages).

**Figure 3:**
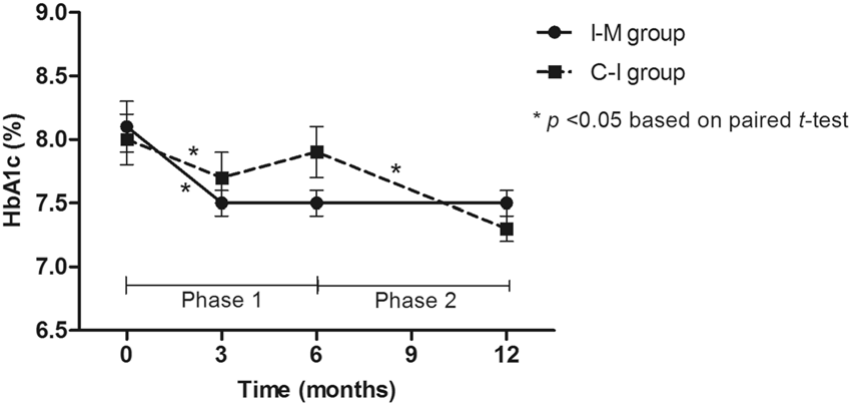
Changes in means and standard errors of glycated hemoglobin (HbA1c) levels over the 12-month study period.

HbA1c levels significantly decreased both in the C-I and I-M groups over the first 3 months (Supplementary Table S2 and Fig. 3). However, improved HbA1c levels were maintained only in the I-M group at 6 months. Decrement of HbA1c levels in the I-M group was 0.6% (from 8.1 ± 1.5 to 7.5 ± 1.1, *P* value < 0.001 based on a paired *t-*test), whereas there was no significant change in the C-I group. Regarding the other parameters, BMI and BP showed significant improvements in both the I-M and C-I groups, and TC and LDL-C levels regressed in the C-I group during the 6-month phase 1 of the study (Supplementary Table S2).

Table 1 shows the changes in diabetes self-management levels and awareness of diabetes estimated from SDSCA and ADS scores. Diabetes self-management showed significant improvements, especially in exercise frequency, SMBG, and number of cigarettes in the I-M group only and foot care in both groups. In addition, after 6 months, the ADS scores showed that participants in the I-M group tended to make more positive appraisals regarding sense of self-control than subjects in the C-I group.

**Table 1:** Changes in SDSCA and ADS Scores in the Groups.

	C-I group	I-M group	*P* value^a^
**SDSCA**			
General diet			
Baseline	2.6 ± 1.8	2.8 ± 2.2	0.61
6 months (Phase 1)	2.7 ± 1.9	3.0 ± 1.7	0.32
12 months (Phase 2)	3.3 ± 1.6^b,c^	3.5 ± 2.0	0.48
Specific diet			
Baseline	2.5 ± 1.1	2.5 ± 1.2	0.92
6 months (Phase 1)	2.8 ± 1.0	2.8 ± 1.1	0.96
12 months (Phase 2)	2.6 ± 1.0	3.0 ± 1.2^b^	0.09
Diet total			
Baseline	2.6 ± 1.3	2.7 ± 1.5	0.73
6 months (Phase 1)	2.8 ± 1.2	2.9 ± 1.1	0.48
12 months (Phase 2)	2.9 ± 1.1^b^	3.3 ± 1.2^b^	0.21
Exercise			
Baseline	3.0 ± 1.6	2.9 ± 1.8	0.90
6 months (Phase 1)	2.9 ± 1.4	3.5 ± 1.8^b^	0.049
12 months (Phase 2)	3.5 ± 1.8^c^	3.1 ± 1.7	0.25
SMBG			
Baseline	1.6 ± 2.2	1.7 ± 2.2	0.72
6 months (Phase 1)	1.9 ± 2.2	3.5 ± 2.3^b^	<0.001
12 months (Phase 2)	3.2 ± 2.3^b,c^	2.6 ± 2.4^b,c^	0.14
Foot care			
Baseline	1.5 ± 2.0	1.5 ± 1.8	0.77
6 months (Phase 1)	2.5 ± 2.1^b^	3.1 ± 2.5^b^	0.21
12 months (Phase 2)	3.0 ± 2.4^b^	3.4 ± 2.5^b^	0.48
Smoking			
Baseline	1.8 ± 3.0	2.1 ± 3.2	0.63
6 months (Phase 1)	1.8 ± 3.0	1.8 ± 3.0	0.91
12 months (Phase 2)	2.2 ± 3.3	1.7 ± 2.9	0.39
Number of cigarettes			
Baseline	3.5 ± 7.2	4.5 ± 8.0	0.48
6 months (Phase 1)	2.3 ± 5.7	3.3 ± 6.8^b^	
12 months (Phase 2)	4.5 ± 7.8	3.4 ± 7.0^b^	
**ADS**			
Psychological impact of diabetes			
Baseline	10.9 ± 2.9	11.1 ± 3.3	0.66
6 months (Phase 1)	10.2 ± 2.8	10.9 ± 3.1	0.20
12 months (Phase 2)	10.8 ± 2.9	10.7 ± 3.2	0.91
Sense of self-control			
Baseline	5.0 ± 1.2	4.7 ± 1.2	0.15
6 months (Phase 1)	4.8 ± 1.3	4.3 ± 1.1^b^	0.02
12 months (Phase 2)	4.7 ± 1.3	4.2 ± 1.0	0.08
Total			
Baseline	18.9 ± 3.3	18.6 ± 4.2	0.70
6 months (Phase 1)	17.9 ± 3.3	17.9 ± 3.3	0.70
12 months (Phase 2)	18.5 ± 3.5	17.8 ± 3.8	0.74

*SDSCA* Summary of the Diabetes Self-Care Activities Questionnaire, *ADS* Appraisal of Diabetes Scale.

^a^*P* values were derived from Student’s *t*-test.

^b^*P* < 0.05, vs. baseline based on Wilcoxon signed rank test.

^c^*P* < 0.05, vs. 6 months based on Wilcoxon signed rank test.

### Phase 2 results

Phase 2 of the study included 112 participants, 105 of whom completed the study (Fig. 2, the numbers of I-M and C-I group were 54 and 51 subjects, respectively). The dropout rate was 6.25% (7 of 112 participants were lost to follow-up). During the 6-month phase 2, 53 participants of the C-I group received approximately 112.3 ± 23.9 app messages (10.0 ± 4.3 messages of tailored feedback and 100.4 ± 24.0 automated messages), and 51 participants of the I-M group received approximately 8.6 ± 3.0 automated app messages.

Table 2 and Fig. 3 show the changes in biochemical and anthropometric parameters throughout the entire study period. HbA1c levels of the C-I group who received TMC during phase 2 of the study decreased by 0.6% compared to phase 1 levels (from 7.9 ± 1.5 to 7.3 ± 1.0, *p* value < 0.001 based on a paired *t-*test). In the I-M group, initial improvement in HbA1c levels at 3 months continued until 12 months. Consequently, HbA1c levels in both the C-I and I-M groups decreased significantly compared to baseline values over the 12-month study period. The mean decrements were 0.8 ± 0.9 and 0.7 ± 1.1, respectively (both *p* values were <0.001 based on paired *t*-tests).

**Table 2:** Changes in Biochemical and Anthropometric Parameters in the Groups over 12 Months.

Variables	C-I group	I-M group	*P* value^a^
HbA1c			
Baseline	8.0 ± 1.2	8.1 ± 1.5	0.59
6 months (Phase 1)	7.9 ± 1.5	7.5 ± 1.1^b^	0.09
12 months (Phase 2)	7.3 ± 1.0^b,c^	7.5 ± 1.1^b^	0.33
BMI (kg/m^2^)			
Baseline	26.3 ± 3.2	26.1 ± 3.3	0.65
6 months (Phase 1)	25.7 ± 3.3^b^	25.7 ± 3.4^b^	0.11
12 months (Phase 2)	26.1 ± 3.1^b^	26.0 ± 3.5^c^	0.20
Systolic BP (mmHg)			
Baseline	138.8 ± 16.1	137.1 ± 15.9	0.53
6 months (Phase 1)	119.7 ± 11.2^b^	120.3 ± 10.4^b^	0.36
12 months (Phase 2)	129.8 ± 13.5^b,c^	128.1 ± 12.3^b,c^	0.72
Diastolic BP (mmHg)			
Baseline	86.6 ± 9.6	87.0 ± 10.4	0.81
6 months (Phase 1)	79.2 ± 7.4^b^	78.6 ± 7.9^b^	0.64
12 months (Phase 2)	83.8 ± 10.2^b,c^	82.4 ± 9.1^b,c^	0.78
Total cholesterol (mg/dL)			
Baseline	168.0 ± 39.5	169.9 ± 32.5	0.76
6 months (Phase 1)	182.7 ± 43.4^b^	171.0 ± 37.9	0.10
12 months (Phase 2)	159.2 ± 38.1^c^	171.4 ± 39.4	0.11
Triglycerides (mg/dL)			
Baseline	165.0 ± 94.7	147.1 ± 61.1	0.20
6 months (Phase 1)	178.8 ± 101.9	140.6 ± 57.9	0.01
12 months (Phase 2)	150.0 ± 98.6^c^	145.1 ± 82.1	0.79
HDL-C (mg/dL)			
Baseline	48.0 ± 14.9	47.3 ± 11.2	0.79
6 months (Phase 1)	48.7 ± 12.2	48.7 ± 10.4	0.99
12 months (Phase 2)	50.3 ± 13.8	51.2 ± 13.4^b^	0.72
LDL-C (mg/dL)			
Baseline	87.1 ± 38.1	93.2 ± 30.0	0.31
6 months (Phase 1)	98.3 ± 41.0^b^	94.2 ± 33.8	0.54
12 months (Phase 2)	79.8 ± 28.8^c^	91.8 ± 35.5	0.06

Data are presented as mean ± standard deviation.

*HbA1c* glycosylated hemoglobin, *BMI* body mass index, *BP* blood pressure, *HDL-C* high-density lipoprotein cholesterol, *LDL-C* low-density lipoprotein cholesterol.

^a^*P* values were derived from Student’s *t*-test.

^b^*P* < 0.05, vs. baseline based on paired *t*-test.

^c^*P* < 0.05, vs. 6 months based on paired *t*-test.

Although the initial improvements of BMI and BP were attenuated during phase 2 in both groups, significant improvements in BP compared with baseline values tended to be maintained, and participants in the C-I group showed a mild decrease in BMI after 12 months (Table 2). The lipid profile and TC, TG, and LDL-C levels significantly decreased during the next 6 months in the C-I group. In addition, HDL-C levels significantly increased in the I-M group compared with baseline.

Regarding diabetes self-management, similar with participants in the I-M group in phase 1 of the study, frequency of exercise and SMBG in the C-I group significantly increased during the intervention period (Table 1). In addition, the frequency of utilizing a general diet in the C-I group and a special diet in the I-M group increased. Consequently, frequency of healthy eating improved in both groups at the end of the 12-month study.

### Adverse events

During the 12-month study period, 4 subjects were hospitalized due to diagnosis of cancer or surgery not related to this study. One participant in the I-M group died from cerebral infarction during phase 2 of the study. There was no change in medication during the study period (Supplementary Table S3). The frequencies of hypoglycemia during the 6-month intervention period in the I-M and C-I groups were 1.1 ± 3.7 and 1.5 ± 3.6, respectively.

## Discussion

In this study, we evaluated the effect of TMC in diabetes care when added to the usual current diabetes management and investigated whether this effect was repeatable in other participants and maintained without active intervention. After 6 months, HbA1c levels were significantly decreased by 0.6%. This improvement was reproduced in the other group of participants and remained after removal of individualized message feedback.

Numerous studies have indicated that digital healthcare systems can be helpful in diabetes management regardless of age, diabetes type, or baseline HbA1c^13–18^. In a multicenter, randomized clinical trial (RCT) conducted in 163 type 2 diabetes patients for one year, mobile coaching that included a mobile application and a web portal resulted in greater decrease in mean HbA1c than usual management. Mean decline of HbA1c was more than 1.0% in the intervention group, regardless of baseline HbA1c levels^13^. In a secondary analysis^15^, when the participants were divided by age of 55 years, the effect of the mobile diabetes-coaching intervention was similar in both groups. In another six-month RCT conducted in 144 Korean type 2 diabetic patients aged >60 years, HbA1c levels were significantly decreased in the intervention group (mean decline of HbA1c was about 0.4%), and the proportion of patients achieving HbA1c <7% without hypoglycemia was greater than in the usual care group^14^. Most of the previous studies were based on automatic text messages immediately sent according to uploaded data^13,14,27,28^, unidirectional advice^14,16^, required additional devices for data transmission^14,16^, provided web portal service as well as mobile application to the participants^13,17^, and had no impact on other clinical outcomes^13,17,19^.

Recently, Young *et al*. reported that an automated one-way alarm via meter did not show any benefit in glycemic control in patients with non-insulin-treated type 2 diabetes at 1 year, although transient improvement of HbA1c was seen at 6 months^29^. The authors mentioned limitations of one-way feedback and requirement of interaction between providers and participants in the durability of this messaging.

Conversely, the TMC system tested in this study provided friendly medical advice including interpretation of uploaded data and provided the opportunity for bidirectional communication between providers and participants without any additional equipment for data transmission or web portal for users. This personalized education was known as a feature of diabetes mobile application^30^. During the 6-month intervention period, the participants in the I-M and C-I groups asked about technical problems and medical issues using the Switch application (7.7 ± 10.6 and 6.1 ± 6.1 times, respectively). This bidirectional communication may have contributed to the positive clinical outcomes^31^.

Considering the mean ages of the C-I and I-M groups (52.6 ± 7.9 and 51.4 ± 7.9, respectively), target HbA1c level was less than 6.5~7.0%^20,21^. Although final HbA1c levels, which were 7.3 ± 1.0 and 7.5 ± 1.1, respectively, did not achieve the desired level, the majority of subjects did not undergo change in antidiabetic medication during the study period (Supplementary Table S3). Predictable HbA1c decrement due to structured exercise training, dipeptidyl peptidase 4 inhibitor, GLP-1 agonists, and thiazolidinedione has been shown to be as high as 0.67%, 0.5–0.8%, 0.5–1.5%, and 0.5–1.4%, respectively^32,33^. Thus, our result implies an effect of tailored mobile coaching in glycemic control. The degree of improvement in HbA1c was moderate compared with previous trials^13,14,16–19^. According to a systemic review, mean decrement of HbA1c in digital healthcare for diabetes was −0.27%^34^. While subjects with mean baseline HbA1c levels ≥9.0% showed approximately 1.0% decreases in HbA1c levels^13,18^, those with a mean baseline level <8.0% showed a smaller decrease in HbA1c levels (approximately 0.4%)^14,16^. In the present study, the proportion of participants with baseline HbA1c levels less than 7% was 12.2% in both groups, and the mean baseline HbA1c was approximately 8.0%. Consequently, a decline in HbA1c levels of 0.6% was predicted.

In addition, the improvements in diabetes self-management, especially regarding SMBG, exercise, and knowledge of diabetes management observed in the present study are notable (Table 1). The wearable fitness tracker might be a strong motivator for patients to exercise more^35–38^. Although increased exercise was transient during the intervention period, this result was comparable with previous analyses showing a high degree of discontinuation after 6 months^39,40^.

During phase 2 of study, the C-I group showed similar improvements to the I-M group, demonstrating universal effectiveness of this TMC system. In addition, another purpose of the maintenance period ofthe I-M group was to investigate whether participants could maintain therapeutic lifestyles learned from individualized mobile coaching during the first 6 months, without supervision, as the residual effect of TMC would not be eliminated, despite the crossover design. As a result, the HbA1c levels in the I-M group were not increased after phase 2 (Table 2). Apparently, the TMC was helpful in successfully educating participants regarding therapeutic lifestyle modifications as well as providing close and consistent supervision of therapeutic lifestyles. Presumably, the participants became more aware of their daily behaviors. However, considering that the subjects were not a healthy population but patients with diabetes, involvement of healthcare professionals is essential to optimize tailored mobile coaching rather than using a non-validated automated feedback system^41,42^. In our study, 3 healthcare professionals attended to 136 participants for 12 months. When intensity of administration is stratified according to health status and motivation, and the TMC system is designed to consist of an intensive coaching period and a self-application period, administration to more participants would be possible. Compared with previous studies regarding mobile healthcare systems, a lower dropout rate was observed^13,14,17,43^. Therefore, this model could be commercially applicable to world-wide users.

Regarding the transient decreases in BMI and BP in 6 months followed by increase by 12 months in both groups, the results should be interpreted cautiously. Transient improvement of weight and BP during the first 6 months seemed to be related to motivation of the participants, who were motivated by offline education before randomization. Attenuation of the weight loss and BP benefits observed at 12 months is consistent with previous interventions demonstrating that maintenance of weight and BP control is more challenging than initial weight reduction^44,45^. In spite of aggravation, there was significant decrement in BP compared with baseline values in both groups. With regard to BMI, the C-I group undergoing voluntary lifestyle modification followed by tailored mobile support showed significant improvement. This result suggests the possibility of digital healthcare systems as a modality for promoting long-term maintenance of healthy habits^46^.

To our knowledge, this is the first trial that investigated the reproducibility and durability of TMC intervention for diabetes management. Nevertheless, this study had several limitations. First, participant digital proficiency and comprehension of our TMC system was not considered. Second, we could not confirm medication adherence. Given that mobile text messaging has been shown to increase medical adherence^47^, medication compliance in our study participants might have been increased. Third, the study population may also be non-representative because they were policyholders of a specific healthcare insurance company. Finally, the presence of complications related to diabetes was not identified. However, the duration of the present study was only 1 year; therefore, development of complications during this study period was likely caused by poor glycemic control before enrollment in this study rather than that developed during the follow-up period.

## Conclusions

In this prospective study, the addition of TMC to conventional diabetes management was shown to be effective in reducing HbA1c levels and improving diabetes self-management. The results were reproducible in another population group and durable without active intervention. This form of digital healthcare system is expected to contribute to the prevention and management of many chronic diseases including diabetes.

## Electronic supplementary material

Supplementary Figure and Tables
