# Supplementary material for: The effectiveness, reproducibility, and durability of tailored mobile coaching on diabetes management in policyholders: A randomized, controlled, open-label study
Source: Sci Rep. 2018 Feb 26;8:3642. doi: 10.1038/s41598-018-22034-0 (PMC5827660; doi:10.1038/s41598-018-22034-0)
Supplement: Supplementary file 1 — Supplementary Figure and Tables [file 41598_2018_22034_MOESM1_ESM.pdf]

The effectiveness, reproducibility, and durability of tailored mobile coaching on diabetes management in policyholders: A randomized, controlled, open-label study

Da Young Lee[1,2], Jeongwoon Park[3], Dooah Choi[3], Hong-Yup Ahn[4], Sung-Woo Park[1], Cheol-Young Park[1]

[1]Division of Endocrinology and Metabolism, Department of Internal Medicine, Kangbuk Samsung Hospital, Sungkyunkwan University School of Medicine, Seoul, Republic of Korea

[2]Division of Endocrinology and Metabolism, Department of Internal Medicine, Korea University College of Medicine, Seoul, Republic of Korea

[3]Huraypositive Inc. Sinsa-dong, Gangnam-gu, Seoul, Republic of Korea

[4]Department of Statistics, Dongguk University-Seoul, Seoul, Republic of Korea

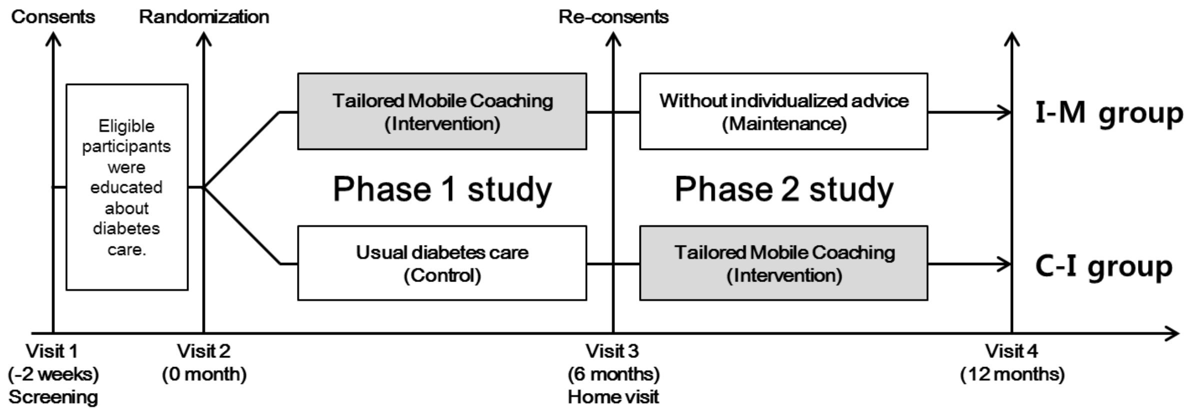

Supplementary Figure S1. Study Design

Variables	C-I group (n=64)	I-M group (n=72)	*P* value[a]
Age (years)	52.6 ± 7.9	51.4 ± 7.9	0.412
Sex, male (%)	44 (68.8)	42 (58.3)	0.219
BMI (kg/m^2)	26.3 ± 3.2	26.1 ± 3.3	0.653
≥ 25.0 kg/m^2	41 (64.1)	41 (56.9)	0.483
Systolic BP (mmHg)	138.8 ± 16.1	137.1 ± 15.9	0.531
Diastolic BP (mmHg)	86.6 ± 9.6	87.0 ± 10.4	0.807
HbA1c (%)	8.0 ± 1.2	8.1 ± 1.5	0.592
6.6-7.0%, n (%)	13 (20.3)	12 (16.7)	0.435
7.1-8.5%, n (%)	34 (53.1)	46 (63.9)	0.435
≥ 8.6%, n (%)	17 (26.6)	14 (19.4)	0.435
Fasting plasma glucose (mg/dl)	175.9 ± 47.7	172.1 ± 46.8	0.642
Total cholesterol (mg/dL)	168.0 ± 39.5	169.9 ± 32.5	0.761
Triglycerides (mg/dL)	165.0 ± 94.7	147.1 ± 61.1	0.199
HDL-C (mg/dL)	48.0 ± 14.9	47.3 ± 11.2	0.786
LDL-C (mg/dL)	87.1 ± 38.1	93.2 ± 30.0	0.306
Current smoker (%)	18 (28.1)	21 (29.2)	0.893
Current alcohol drinker (%)	38 (63.3)	30 (42.3)	0.022
Duration of diabetes (years)	9.3 ± 6.0	7.5 ± 4.8	0.060
Insulin injection (%)	12 (19.7)	16 (22.2)	0.719
Antihypertensive medication (%)	29 (47.5)	27 (37.5)	0.386
Antidyslipidemic medication (%)	30 (49.2)	38 (52.8)	0.607
SDSCA			
Diet			

General diet (times/week)	2.5 ± 1.8	2.9 ± 2.2	0.324
Special diet (times/week)	2.5 ± 1.1	2.6 ±1.2	0.777
Total (times/week)	2.5 ± 1.2	2.7 ±1.5	0.474
Exercise (times/week)	3.0 ± 1.7	2.9 ±1.8	0.634
Blood-glucose testing (times/week)	1.5 ± 2.2	1.8 ±2.2	0.580
Foot care (times/week)	1.5 ± 2.0	1.6 ±1.8	0.879
Smoking (times/week)	1.9 ± 3.0	2.0 ± 3.1	0.924
Amount (cigarettes/day)	3.8 ± 7.4	4.3 ± 7.8	0.728
ADS			
Psychological impact of diabetes	10.7 ± 3.0	11.1 ± 3.3	0.455
Sense of self-control	5.0 ± 1.2	4.7 ± 1.2	0.079
Total	18.8 ± 3.3	18.6 ± 4.3	0.824

Supplementary Table S1. Baseline Characteristics of Participants by Group

Data are presented as mean ± standard deviation or as number (%).

BMI body mass index, *BP* blood pressure, *HbA1c* glycosylated hemoglobin, *HDL-C* high-density lipoprotein cholesterol, *LDL-C* low-density lipoprotein cholesterol, *SDSCA* Summary of the Diabetes Self-Care Activities Questionnaire, *ADS* Appraisal of Diabetes Scale.

[a]*P* values were derived from Student’s t-test and chi-square test.

Variables	C-I group (n=64)	I-M group (n=72)	*P* value[a]
HbA1c			
Baseline	8.0 ± 1.2	8.1 ± 1.5	0.59
3 Months	7.7 ± 1.3	7.5 ± 1.0	0.21
P value *vs*. baseline	0.03	< 0.001	
6 Months	7.9 ± 1.5	7.5 ± 1.1	0.09
P value *vs*. baseline	0.46	< 0.001	
BMI (kg/m^2)			
Baseline	26.3 ± 3.2	26.1 ± 3.3	0.65
3 Months	25.9 ± 3.3	25.7 ± 3.4	0.74
P value *vs*. baseline	0.01	< 0.001	
6 Months	25.7 ± 3.3	25.7 ± 3.4	0.11
P value *vs*. baseline	< 0.001	0.01	
Systolic BP (mmHg)			
Baseline	138.8 ± 16.1	137.1 ± 15.9	0.53
3 Months	122.8 ± 11.2	122.2 ± 11.7	0.74
P value *vs*. baseline	< 0.001	< 0.001	
6 Months	119.7 ± 11.2	120.3 ± 10.4	0.36
P value *vs*. baseline	< 0.001	< 0.001	
Diastolic BP (mmHg)			
Baseline	86.6 ± 9.6	87.0 ± 10.4	0.81
3 Months	81.3 ± 6.0	79.7 ± 6.9	0.15
P value *vs*. baseline	< 0.001	< 0.001	

6 Months	79.2 ± 7.4	78.6 ± 7.9	0.64
P value *vs*. baseline	< 0.001	< 0.001	
Total cholesterol			
Baseline	168.0 ± 39.5	169.9 ± 32.5	0.76
3 Months	181.2 ± 42.0	176.8 ± 37.8	0.44
P value *vs*. baseline	0.01	0.05	
6 Months	182.7 ± 43.4	171.0 ± 37.9	0.10
P value *vs*. baseline	0.01	0.77	
Triglycerides			
Baseline	165.0 ± 94.7	147.1 ± 61.1	0.20
3 Months	167.8 ± 106.6	141.0 ± 64.0	0.08
P value *vs*. baseline	0.79	0.41	
6 Months	178.8 ± 101.9	140.6 ± 57.9	0.01
P value *vs*. baseline	0.27	0.41	
HDL-C			
Baseline	48.0 ± 14.9	47.3 ± 11.2	0.79
3 Months	47.6 ± 12.5	46.9 ± 12.0	0.73
P value *vs*. baseline	0.76	0.63	
6 Months	48.7 ± 12.2	48.7 ± 10.4	0.99
P value *vs*. baseline	0.62	0.11	
LDL-C			
Baseline	87.1 ± 38.1	93.2 ± 30.0	0.31
3 Months	101.0 ± 37.0	101.7 ± 34.1	0.91
P value *vs*. baseline	< 0.001	0.01	

6 Months	98.3 ± 41.0	94.2 ± 33.8	0.54
P value *vs*. baseline	0.01	0.75	

Supplementary Table S2. Changes in Biochemical and Anthropometric Parameters in the Groups during Phase 1 of the Study

Data are presented as mean ± standard deviation or as number (%).

HbA1c glycosylated hemoglobin, *BMI* body mass index, *BP* blood pressure, *HDL-C* high-density lipoprotein cholesterol, *LDL-C* low-density lipoprotein cholesterol.

[a]*P* values were derived from Student's *t*-test or paired *t*-test.

	C-I group	I-M group	*P* value[a]
Phase 1 study (n=136)	n=64	n=72	
Dose up antidiabetic medication	8 (12.5)	14 (19.4)	0.485
Dose down antidiabetic medication	4 (6.3)	7 (9.7)	0.754
Dose up antihypertensive medication	2 (3.1)	7 (9.7)	0.150
Dose down antihypertensive medication	2 (3.1)	3 (4.2)	0.999
Dose up antidyslipidemic medication	4 (6.3)	5 (6.9)	0.999
Dose down antidyslipidemic medication	1 (1.6)	4 (5.6)	0.381
Phase 2 study (n=105)	n=51	n=54	
Dose up antidiabetic medication	8 (15.7)	8 (14.8)	0.999
Dose down antidiabetic medication	8 (15.7)	5 (9.3)	0.375
Dose up antihypertensive medication	1 (2.0)	0 (0)	0.999
Dose down antihypertensive medication	0 (0)	3 (5.6)	0.232
Dose up antidyslipidemic medication	4 (7.8)	3 (5.6)	0.703
Dose down antidyslipidemic medication	1 (2.0)	5 (9.3)	0.197

Supplementary Table S3. Changes of Medication by Group

[a]*P* values were derived from Chi-square test.
